# Insights on poster preparation practices in life sciences

**DOI:** 10.3389/fbinf.2023.1216139

**Published:** 2023-11-01

**Authors:** Helena Klara Jambor

**Affiliations:** National Center for Tumor Diseases, University Cancer Center, NCT-UCC, Technische Universität Dresden, Dresden, Germany

**Keywords:** BioVis, data visualization, science communication, visual communication, data viz, poster design, science education, infographics

## Abstract

Posters are intended to spark scientific dialogue and are omnipresent at biological conferences. Guides and how-to articles help life scientists in preparing informative visualizations in poster format. However, posters shown at conferences are at present often overloaded with data and text and lack visual structure. Here, I surveyed life scientists themselves to understand how they are currently preparing posters and which parts they struggle with. Biologist spend on average two entire days preparing one poster, with half of the time devoted to visual design aspects. Most receive no design or software training and also receive little to no feedback when preparing their visualizations. In conclusion, training in visualization principles and tools for poster preparation would likely improve the quality of conference posters. This would also benefit other common visuals such as figures and slides, and improve the science communication of researchers overall.

## Introduction

Visualizations are important to communicate biological and medical sciences since antiquity (Stückelberger, 1994). Today, scientists visually present biomedical data in manuscript figures and, at least since the 1970s, with scientific posters at conferences (Maugh, 1974; Dubois, 1985a). Posters are a large-scale visualizations juxtaposed of explanatory text with pictures, schematics/diagrams, and/or data visualizations. Poster presentation sessions are popular events (Rowe, 2017), especially so when participant numbers exceed the speaker slots. At large meetings thousands of posters may be presented: 2,500 at the meeting of the American Society for Cell Biology, 3,000 at the meeting of the American Society for Human Genetics, and 1,500 at the meeting of Technology Association of Grantmakers (TAG). Posters are particularly popular among early career researchers, who use posters to show in-progress projects and have personal discussions. For many early career scientists, a poster session is their first experience in publicly presenting results to colleagues and a wider audience.

Posters are common also beyond life science and medicine: posters and poster presentations in physics, law, and clinical psychology share similarities in their written and visual approaches (D’Angelo, 2016) and posters in linguistics, chemistry, and speech and hearing research also were found to have comparable proportions of visuals and text (Li, 2014). Both studies found that natural science posters overall had less text and a higher focus on visual representations.

Across life sciences, posters include a header with title and author information, an overview of the rationale, methods, and results, and at times references, acknowledgements, and summaries (Dubois, 1985b; Brown, 1996). This general poster structure is described as the “IMRAD format” and echoes the organisation of a scientific manuscript, with a title including the authors followed by introduction, oftentimes the text submitted as abstract, a brief methodology section, representative results, and a brief discussion (Brown, 1996). The layout dictates the order in which the elements of the posters should be read and must be well designed by the author (Dubois, 1985b). At poster sessions, scientists should provide audiences a poster presentation, a brief narration of the entire poster also referred to as the “elevator pitch” (Erren and Bourne, 2007). The poster and its presentation is a form of visually-aided storytelling.

Several guidelines help life scientists in preparing legible and concise posters (Brown, 1997, 1996; Block, 1996; Erren and Bourne, 2007; Fraser et al., 2016; Barker and Phillips, 2021; Faulkes, 2021; Wang et al., 2022). These guidelines are explicitly written for scientists with little or no previous knowledge in visual design and low visualization literacy. Boullata and Mancuso (2007); Gundogan et al. (2016) provide updates with an exemplar template, details on text, font size, information flow and the importance of feedback. Erren and Bourne also provide recommendations for the oral presentation and suggest to having an “elevator pitch” ready. In addition, there is also a growing body of literature helping life scientists with the overall presentation of visual information. Bang Wong initiated a long-running series of articles that cover many aspects of design and visualization principles that are relevant also to posters or to the elements within a poster: these articles comment on the elements of visual style, layout and Gestalt principles, but also colours and storytelling (Wong, 2011a, 2011b, 2011c; Krzywinski, 2013; Krzywinski and Cairo, 2013). Importantly, the intended audiences are early career life scientists struggling with visual design of information. Helping biologists further, many universities already provide poster templates for standard software that have a pre-set layout and new tools such as Biorender have ready-to-go poster templates.

Despite these helpful resources, not all posters are well-designed to be easily understandable. A commentary recounts all that can go wrong with poster presentations, such as too much text, cryptic abbreviations, inconsistent colours, and a lack of visual order (Wolcott, 1997). A recent study quantified this: the authors reveal that the format of award-winning posters at life science conferences still adheres to the standard format, with the majority, 75%, of posters using the “Introduction, methods, results, discussion” format and are heavy on text, colloquially referred to as the “wall of text” (Faulkes, 2023). The author also demonstrated that at least 30% of these pre-selected, award winning posters had no clear reading orientation, and overall less than 50% of the posters followed good graphic design practices (Faulkes, 2023). Given the plethora of guides for poster preparation, it remains an open question as to why the quality of posters at conferences is often poor. In particular, as only award-winning posters were included in the quantitative study, it stands to reason that the majority of posters have even lower overall appeal (Faulkes, 2023). Where in the poster preparation, a complex and challenging visualization format, are biological scientists struggling? Do early career scientists receive training in this common visualization format? Are they familiar with existing guidelines and helpful articles?

Here we used a qualitative survey and in-person interviews to shed light on the subjective perspective of life scientists and their poster preparation. Using an observational, anonymous, survey-based approach, we asked life scientists about their time commitment towards poster preparation, including the amount of time spend on design aspects. We also enquired on previous training they had received and the software and design process towards preparing this visual format. Our data thus summarises the challenges faced by likely many more scientists and may thus help in orienting readers towards preparing a suitable training for early career scientists.

## Methods

### Participants

This work is based on 1) an anonymous online survey data from 90 participants, and on 2) 23 participants from an in-person interview survey. No personal data was collected from the voluntary respondents. Prior to the interview/online survey, participates were informed that anonymous answers from the survey would be used for a summarising review of poster design practices, that participation was voluntary, that they could withdraw any time, and whom to reach for further questions. The informed consent was provided to interviewees and survey respondents. The 23 in-person interviews were conducted at international life science conference in Germany, and the online survey was circulated via social media and by Email.

### Questions of the online survey

Country of studies, Subject of studies, Previous training in poster making, Number of posters prepared, Time spend on poster preparation, Time spend on poster design, Number of people involved in poster preparation/their role, Poster designed from scratch/template.

### Questions of the in-person interview

Gender (Male/female, categorical), Career stage (Predoc/Postdoc/PI, categorical), Country of studies, Subject of studies, Time for poster preparation (<1 h, 1 day, >1 day, categorical), Time spend on poster design (none, <1 h, > 1 h, categorical), Software used for poster preparation, Number of people involved in poster preparation/their role, Poster designed from scratch/template, Previous training in poster making, Personal critique of poster (what went well, what was difficult/frustrating).

### Data analysis

Data were summarised (Supplementary Table S1) with descriptive statistical methods and analysed with the statistical software R, figures were prepared with a vector graphic software.

## Results

### Online user survey

**FIGURE 1:**
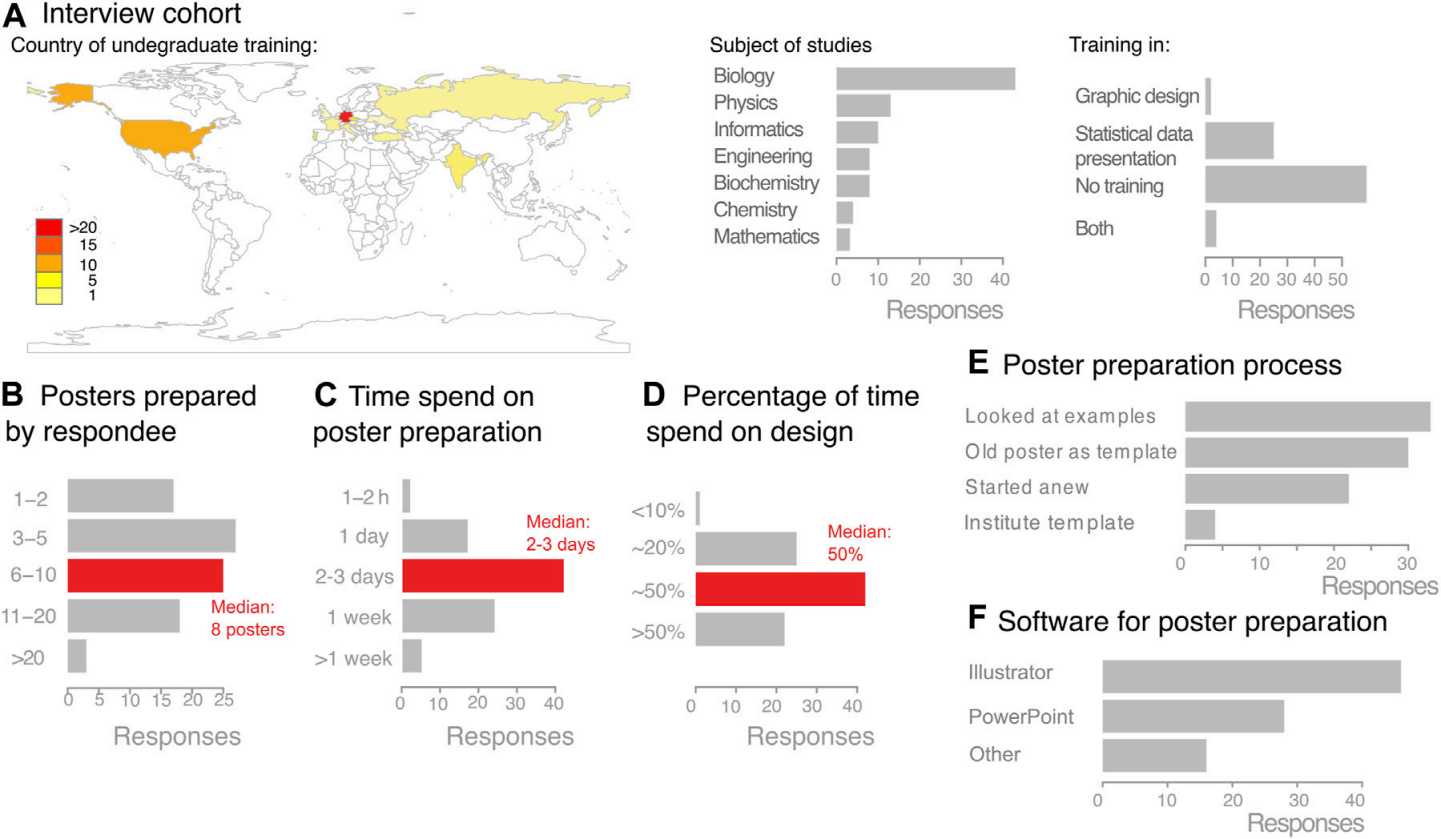
Online survey on the poster preparation process among life scientists. **(A)** Interview cohort composition. **(B–F)**. Collected responses on the poster preparation process of online survey participants.

In an anonymous online survey we asked scientists working in biological and medical research 9 questions about their poster preparation practices (Supplementary Table S1. The online survey included responses 90 scientists who had received their undergraduate training in 26 countries (Figure 1A). Around half of the group had studied biology/related subjects, the other half included physicist, engineers, chemists and computer scientists working in biological or medical research (Figure 1A). 25 had received some training in statistical data representation, 2 respondents reported training graphical presentation of data and/or design principles for information design, and 4 had received training in both subjects (Figure 1A).

During their academic careers, respondents had already prepared a median of 8 posters (Figure 1B). They self-estimated that each poster took two to three working days in preparation time, with around half of this time, one to one-and-a-half days, exclusively devoted to design aspects (Figures 1C, D). For poster preparation, most of which were prepared without an existing template, 51% of the online polled participants used vector-based graphic software (Adobe Illustrator, CorelDraw, Inkscape) and 31% used PowerPoint (Figures 1E, F). Notably, the majority of those polled had either received no feedback (67%) or only limited feedback from just one co-author (17%)—only 5% had received thorough feedback from all poster authors.

### User interviews

**FIGURE 2:**
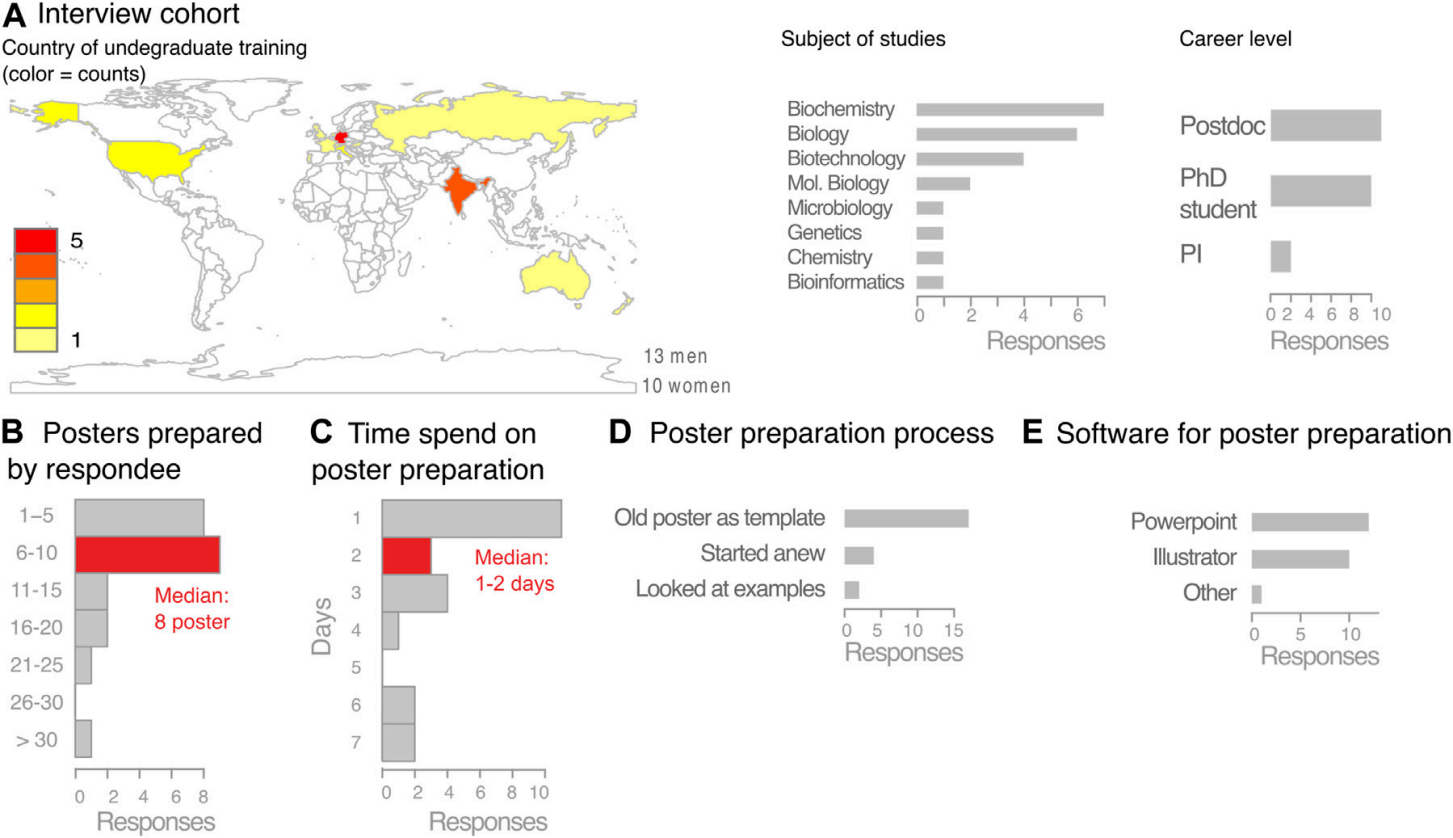
In-person interview with life scientists on their poster preparation processes. **(A)** Interview cohort composition. **(B–E)** Collected responses on the poster preparation process of in-person interviews.

We performed in-person interviews with 23 participants presenting a poster at a molecular biology conference (Figure 2A; Supplementary Table S1). We interviewed around 10% of the total poster presenters and were limited to the poster session times when presenters were found at their poster stands (in total two presentation slots in the course of the conference).

The 23 interviewees included 13 male and 10 female scientists, the majority of which (21/23), had completed undergraduate studies in 15 different countries in biology or a closely related field in various countries (Figure 2A). Only 1 among the 23 interviewees had received training in graphical representation of statistical data. None of the respondent reported to have received training in graphic design principles or poster preparation during either undergraduate or graduate education.

The career stages were diverse with two principal investigators, eleven postdocs, and ten PhD students (Figure 2A). On average the interviewees had prepared around eight posters in their scientific life, but many had prepared substantially more, up to 35 posters (Figure 2B). The median time for preparing the poster was one to two days, with half of the time spend on poster design aspects including layout, colours, and text arrangement/fonts, etc (Figure 2C).

When discussing the process of poster presentation, it emerged that most interviewees (17/23) had not started their poster from scratch, but instead recycled an older poster of theirs or a colleague. In most cases this template was a PowerPoint file, which was also the software most used for poster preparation for 13 participants, while 10 used a vector-graphic software (Figure 2D).

Some interviewees, especially those who had prepared posters with PowerPoint, expressed frustration that images resolution suffered after scaling the slide to poster size (Figure 2E). Correspondingly, several participants stated that they lacked the time to learn using a vector-based software and had not been given a chance for a training in appropriate software. One person stated that the poster process took almost a week because they used the poster preparation as a chance to learn Illustrator. Similar to the online responses, the interviewees had usually not received feedback on their poster preparation from either co-workers or their principal investigator.

## Discussion

Guides and resources are published to help life scientists create powerful posters. Posters, however, pose a challenging visualization format to create and are oftentimes not attractive to audiences (Faulkes, 2023). In this perspective we surveyed those preparing posters about their current challenges and approaches when designing scientific posters. This revealed that scientists spend on average two full working days on poster preparation and face obstacles such as a deficit in training and lack of feedback.

A key observation is the large time commitment for each poster preparation. In addition, respondents report to create around eight posters as a PhD student in biology, which sums up to 14–21 working days spend on poster preparation. These posters are the basis for the first experience of early career researchers in a public scientific conversations. Despite this, poster preparation receives little explicit attention in the training of students and scholars. This lack of training and curricula may in part be due to a lack of insight into the current poster preparation process and its challenges. In addition, the rare feedback from colleagues and supervisors, seems a missed opportunity for training, especially when considering that these visualizations often are used for manuscripts.

While scientists spend considerable amount of time on posters, they and their audiences are still often not satisfied with the results. Lack of knowledge of suitable software and lack of training in data visualization slow down this process and make it prone to errors when visual design principles (Tufte, 1990; Wong, 2011a, 2011b, 2010, 2010; Fraser et al., 2016; Faulkes, 2021) are not applied or when unsuitable software leads to compression artifacts. Life scientist, who in addition to posters also prepare data visualizations for manuscripts or slides, should be trained in software and design fundamentals. While notable books on better posters (Fraser et al., 2016; Faulkes, 2021) and several guidelines for poster preparation and presentation have been published (Brown, 1996, 1996; Block, 1996; Erren and Bourne, 2007; Barker and Phillips, 2021; Wang et al., 2022), general guidelines for preparing diagrams and figures exist from publishers (Nature Guidelines, 2023), and articles provide insights into the design process (Wong, 2011d; 2011a; 2011b), these resources seem to not sufficiently reach their target audiences.

At this point one may only speculate as to why resources are not used. First, the subject may not be taught in undergraduate or graduate curricula, when reading materials are widely spread to the next-generation. Second, it is also possible that too few advisors themselves are well informed about science communication. Integrating science communication, including visual communication, e.g., with posters, into the core training of life scientists seems essential for better posters and eventually better overall visual communication of scientific results. Given that conference poster presentations are important in particular for early careers researchers to present their data for the first time in public, improving the poster quality likely will also improve the scientific discussions. And the scale is not insignificant: it is estimated that in total several million conference posters may be presented every year (Rowe, 2017), all of which could benefit from better poster design processes. Thus, designing biological data visualization and its many formats, including posters, should be an essential building block in curricula.

Re-assessing preparation for conferences is timely, given that in-person events are rapidly re-emerging. Innovations established due to COVID restrictions and now the climate challenges additionally spurred innovations in conferences formats, including new forms of poster sessions (Tao et al., 2021; Skiles et al., 2022) and as equity and inclusivity of traditional conference formats are being debated (Sarabipour et al., 2021), more innovations can be expected. Posters are now often presented both in-person and in virtual spaces, accompanied by poster-flash talks, or short recorded talks. The higher exposure expected from virtual poster presentations and short poster videos, often available for longer times than the conference, should be used as an opportunity to also improve the preparation of scientific posters. Biological and medical visualization researcher could and should help to innovate poster presentations, e.g., with a framework for assessing poster design quality or a focus in the “BioVis” conference tracks at IEEE Vis and ISMB (http://biovis.net/). In longer term, such rules could be integrated into data-driven interactive assessments of the visualizations and provide users feedback on their design quality, as it was tested for evaluating colour design practices in visualizations (InfoColorizer, see Yuan et al., 2022). A larger survey of poster design, possibly also a controlled laboratory study instead of self-reported observations, could inform exactly which quality criteria and rules are important to be included in such a tool.

As an immediate actionable measure, senior scientists can effectively improve poster quality by: 1. Providing early career colleagues with an example poster as a starting point for a first conference poster; 2. Establishing a feedback routine that also discusses the visual design of the poster, and 3. Integrating vector-graphic software, e.g., Adobe Illustrator or Inkscape (open source) in core training to obtain print-quality, professional posters. These simple measures, and perspectively also improving training in visualization principles and tools, will improve the quality of poster visualizations at biology conference and benefit the overall science communication efficacy.

**TABLE 1:** Summarized data online survey and in-person interviews.

Question	Answers	Online survey	In-person interview
Number of posters	1–2	17	5
	3–5	27	3
	6–10	25	9
	11–20	18	4
	>20	3	2
Poster time	1–2 h	2	-
	1 day	17	11
	2–3 days	42	7
	1 week	24	5
	>1 week	5	-
Percentage design	<10%	1	8
	∼10%	25	4
	∼50%	42	8
	>50%	22	3
Poster Process	From scratch	22	3
	Looking at examples	33	2
	With a previous template	30	18
	With an institute template	4	0
Software used	Powerpoint	28	12
	Illustrator	46	10
	other	16	1

## Data Availability

The datasets presented in this study can be found in online repositories. The names of the repository/repositories and accession number(s) can be found below: https://doi.org/10.5281/zenodo.8199952.
