# Supplementary material for: Insights on poster preparation practices in life sciences
Source: Front Bioinform. 2023 Nov 1;3:1216139. doi: 10.3389/fbinf.2023.1216139 (PMC10646475; doi:10.3389/fbinf.2023.1216139)
Supplement: Supplementary file 1 [file Table1.DOCX]

**Table 1. Summarized data online survey and in-person interviews.**

| Question | Answers | Online survey | In-person interview |
| --- | --- | --- | --- |
| Number of posters | 1-2 | 17 | 5 |
|  | 3-5 | 27 | 3 |
|  | 6-10 | 25 | 9 |
|  | 11-20 | 18 | 4 |
|  | >20 | 3 | 2 |
| Poster time | 1-2 hours | 2 | - |
|  | 1 day | 17 | 11 |
|  | 2-3 days | 42 | 7 |
|  | 1 week | 24 | 5 |
|  | >1 week | 5 | - |
| Percentage design | <10% | 1 | 8 |
|  | ~10% | 25 | 4 |
|  | ~50% | 42 | 8 |
|  | >50% | 22 | 3 |
| Poster Process | From scratch | 22 | 3 |
|  | Looking at examples | 33 | 2 |
|  | With a previous template | 30 | 18 |
|  | With an institute template | 4 | 0 |
| Software used | Powerpoint | 28 | 12 |
|  | Illustrator | 46 | 10 |
|  | other | 16 | 1 |
